# Diagnostic and Clinical Impact of Imaging Modality on PSA Density: TRUS Versus MRI in Gray-Zone Prostate Cancer

**DOI:** 10.3390/curroncol33040221

**Published:** 2026-04-16

**Authors:** Davut Unsal Capkan, Mehmet Solakhan

**Affiliations:** 1 Department of Radiology, Medical Point Hospital, 27060 Gaziantep, Turkey; 2 Department of Urology, Medical Point Hospital, 27060 Gaziantep, Turkey; msolakhan@hotmail.com

**Keywords:** PSA density, prostate cancer, MRI, TRUS, PI-RADS, diagnostic performance, decision curve analysis

## Abstract

**Background:** In this study, it was aimed to compare transrectal ultrasound (TRUS)- and magnetic resonance imaging (MRI)-derived prostate-specific antigen density (PSAD) in patients with gray-zone PSA levels (4–10 ng/mL), evaluate their diagnostic performance for clinically significant prostate cancer (csPCa), and assess the clinical implications of reclassification across commonly used thresholds. **Methods:** We retrospectively analyzed 202 men who underwent both TRUS and multiparametric MRI between January 2020 and June 2025. Prostate volume was measured using the ellipsoid formula for TRUS and contour-based planimetry for MRI. PSA density (PSAD) was calculated as total PSA (tPSA, ng/mL) divided by prostate volume (mL) for each modality: TRUS-PSAD and MRI-PSAD. Agreement between modalities was evaluated using Bland–Altman plots and correlation analyses. Reclassification at PSAD thresholds of 0.15, 0.20, and 0.30 ng/mL/mL was assessed using Cohen’s κ and net reclassification improvement (NRI). Diagnostic performance for csPCa (ISUP grade group ≥ 2) was evaluated with ROC analysis and the DeLong test. Inter- and intra-observer reproducibility was determined using intraclass correlation coefficients (ICC) and Cohen’s κ. Clinical utility was assessed by decision curve analysis (DCA). **Results:** MRI-derived prostate volumes were significantly lower than TRUS-derived volumes (median 47.0 vs. 52.5 mL, *p* < 0.001), resulting in higher MRI-PSAD values (median 0.14 vs. 0.12 ng/mL/mL, *p* < 0.001). Bland–Altman analysis demonstrated a negative bias for prostate volume (−3.2 mL) and a positive bias for PSAD (+0.03). Strong correlations were observed between TRUS and MRI measurements (r = 0.96 for volume and r = 0.94 for PSAD). MRI-PSAD frequently reclassified patients into higher risk categories, yielding positive net reclassification improvement for cancer cases across all thresholds, while introducing some negative reclassification among non-cancer cases. ROC analysis showed comparable overall diagnostic performance between TRUS-PSAD and MRI-PSAD (AUC 0.681 vs. 0.679, *p* = 0.91). However, MRI-PSAD demonstrated higher sensitivity at predefined thresholds at the expense of reduced specificity, reflecting a threshold-dependent shift rather than improved discrimination. Reproducibility was higher for MRI-derived measurements (ICC = 0.94; κ = 0.83) compared with TRUS (ICC = 0.86; κ = 0.71). Decision curve analysis indicated that MRI-PSAD, particularly when combined with PI-RADS ≥ 3, provided the greatest net clinical benefit at lower threshold probabilities (5–15%). **Conclusions:** MRI-derived PSA density produces systematically higher values than TRUS-based measurements due to inherent differences in prostate volume estimation. While this results in increased sensitivity at standard thresholds, overall discrimination remains unchanged. These findings support the use of modality-specific PSAD thresholds rather than uniform cutoffs across imaging techniques. In clinical practice, MRI-PSAD may provide additional value when interpreted in conjunction with PI-RADS, primarily through improved threshold calibration rather than enhanced diagnostic accuracy.

## Simple Summary

In men with prostate-specific antigen (PSA) levels in the diagnostic gray zone (4–10 ng/mL), determining the need for biopsy remains challenging. PSA density (PSAD), calculated using prostate volume, is widely used to improve risk stratification; however, the imaging modality used for volume measurement may significantly influence PSAD values. In this study, we compared PSAD derived from transrectal ultrasound (TRUS) and magnetic resonance imaging (MRI) in the same patients. We found that MRI-based measurements yielded lower prostate volumes and consequently higher PSAD values, frequently reclassifying patients into higher-risk categories. Although overall diagnostic accuracy was similar between the two methods, MRI-PSAD demonstrated higher sensitivity and better reproducibility. Importantly, combining MRI-PSAD with PI-RADS scoring provided the greatest clinical benefit in biopsy decision-making. These findings suggest that MRI-derived PSAD may improve risk assessment and help clinicians make more accurate and individualized biopsy decisions in patients with gray-zone PSA levels.

## 1. Introduction

Prostate cancer is the second most commonly diagnosed malignancy in men worldwide and constitutes a major cause of cancer-related morbidity and mortality. Early detection is vital, as timely diagnosis and treatment substantially improve outcomes [1,2,3,4,5]. Serum prostate-specific antigen (PSA) testing remains a cornerstone of prostate cancer screening; however, its diagnostic specificity is limited, particularly in the “gray zone” of 4–10 ng/mL. In this PSA range, benign prostatic hyperplasia (BPH) and prostatitis frequently contribute to elevated levels, complicating clinical decision-making [6,7,8,9].

To refine diagnostic accuracy, PSA density (PSAD)—the ratio of PSA to prostate volume—has been introduced as a complementary biomarker. PSAD improves the discrimination between benign and malignant disease and is now recommended by leading guidelines, including those of the European Association of Urology (EAU) and the National Comprehensive Cancer Network (NCCN), as part of risk stratification before biopsy [10,11]. Threshold values between 0.15 and 0.20 ng/mL/mL are widely used; patients exceeding these cutoffs are more likely to harbor clinically significant prostate cancer (csPCa) [12,13,14].

Accurate estimation of prostate volume is essential for reliable PSAD calculation. Transrectal ultrasound (TRUS) is the most commonly applied modality in routine practice owing to its accessibility and low cost. Nevertheless, TRUS measurements are subject to operator dependency and inter-observer variability [15,16,17]. Compared with TRUS, multiparametric magnetic resonance imaging (mpMRI) may provide improved anatomic delineation and more reproducible prostate volume estimation, although the degree of advantage may vary depending on technique and reader experience [6,18,19,20]. Comparative studies have shown that mpMRI-derived prostate volumes more closely approximate pathologic gland size than TRUS-derived volumes [21,22].

These differences in volume assessment directly influence PSAD values. Several investigations demonstrate that mpMRI-based PSAD achieves higher diagnostic accuracy than TRUS-based PSAD [16,23,24]. Aphinives et al. found that MRI-derived PSAD improved sensitivity and specificity for csPCa in patients with gray-zone PSA [25]. Guo et al. confirmed the consistency of MRI volume measurements over TRUS, reinforcing the role of MRI in standardizing PSAD calculation [2], as also demonstrated in recent fusion biopsy-based studies showing significant correlations between mpMRI-derived parameters and Gleason score [26].

Such variability has critical clinical implications. Overestimation of prostate volume may underestimate PSAD, delaying necessary biopsies, whereas underestimation may lead to unnecessary invasive procedures. Both EAU and NCCN guidelines acknowledge PSAD as a valuable parameter to refine biopsy indications, particularly when mpMRI findings are equivocal [27,28].

Given the multiplicity of imaging modalities—including TRUS, mpMRI, and emerging ultrasound techniques—and the evolving thresholds of PSAD, discrepancies in calculated values across methods may lead to divergent clinical decisions, particularly regarding biopsy indication in patients within the gray PSA zone. Therefore, the present study aimed to systematically compare prostate volumes measured by TRUS and mpMRI in the same individuals, quantify the resulting differences in PSAD, and evaluate their clinical implications for biopsy decision-making. We hypothesize that mpMRI-based PSAD may provide more consistent estimates and improved clinical alignment with disease thresholds, thereby enhancing diagnostic confidence, reducing unnecessary procedures, and contributing to the standardization of prostate cancer risk stratification.

## 2. Materials and Methods

### 2.1. Study Design and Population

This retrospective, single-center observational study was conducted at Medical Point Hospital, Gaziantep, Turkey. Ethical approval was obtained from the Gaziantep City Hospital Non-Interventional Clinical Research Ethics Committee (Decision no: 2025/265, date: 16 July 2025). All procedures were performed in accordance with the Declaration of Helsinki. Due to the retrospective design and use of anonymized data, the requirement for written informed consent was waived by the ethics committee.

Male patients aged 40 years and older who presented to the urology outpatient clinic between January 2020 and June 2025 with a preliminary diagnosis of prostate cancer were screened. For the method-comparison objective, sample size targeted precision of Bland–Altman limits-of-agreement (LoA), using prior or pilot estimates of the SD of paired differences in PSAD; calculations were based on the approach of Lu et al. to ensure the 95% CI width around LoA was within a clinically acceptable margin (Δ) with ≥80–90% assurance [29]. For the diagnostic-performance objective, no formal sample size calculation was based on a prespecified superiority margin for the AUC difference between MRI-PSAD and TRUS-PSAD, as both measures were obtained from the same patients and were expected to be highly correlated. Instead, the available cohort was considered sufficient to provide precision-oriented estimates of AUC, sensitivity, specificity, and reclassification across prespecified PSAD thresholds. Accordingly, ROC-based comparisons should be interpreted as exploratory within the context of this retrospective study.

**Figure 1:**
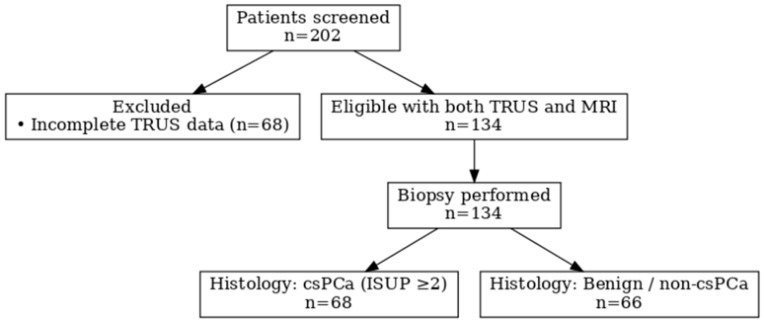
STARD flow diagram of patient selection.

Inclusion criteria were: serum total prostate-specific antigen (PSA) levels between 4 and 10 ng/mL; both transrectal ultrasonography (TRUS) and multiparametric magnetic resonance imaging (mpMRI) performed within a maximum interval of three months; available prostate volume measurements from both modalities; and complete clinical and laboratory data. Exclusion criteria included: PSA values outside the 4–10 ng/mL range, missing or non-interpretable imaging/laboratory data, availability of only one imaging modality, imaging–PSA interval longer than three months, age below 40 or above 70 years, and restricted patient groups such as soldiers, prisoners, or individuals lacking mental competence. PSA eligibility was determined based on values at the time of initial clinical evaluation (Figure 1).

### 2.2. Data Collection and Definitions

**Figure 2:**
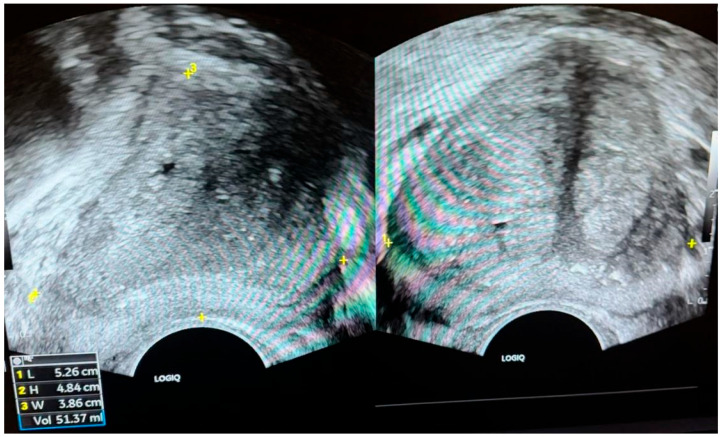
TRUS images of the prostate. (**Left**) Axial image—maximum transverse (width) diameter measured (5.53 cm). (**Right**) Sagittal image—superoinferior (length, 4.68 cm) and anteroposterior (depth, 3.81 cm) diameters measured. Prostate volume calculated as ~50 mL.

**Transrectal ultrasound (TRUS) protocol:** TRUS was performed with a 6–12 MHz end-fire or biplanar transducer by fellowship-trained operators. Prostate dimensions were measured as: transverse (width) and anteroposterior (height) on the axial plane at maximal transverse diameter, and craniocaudal (length) on a mid-sagittal plane slightly off midline to avoid bladder neck foreshortening. Prostate volume was calculated using the prolate-ellipsoid formula: width × height × length × 0.52. When feasible, each dimension was obtained in triplicate and averaged. Operators were blinded to MRI volumes and pathology. Prostate volume was calculated using the prolate-ellipsoid formula based on transverse, superoinferior, and anteroposterior diameters, as illustrated in Figure 2.

**Figure 3:**
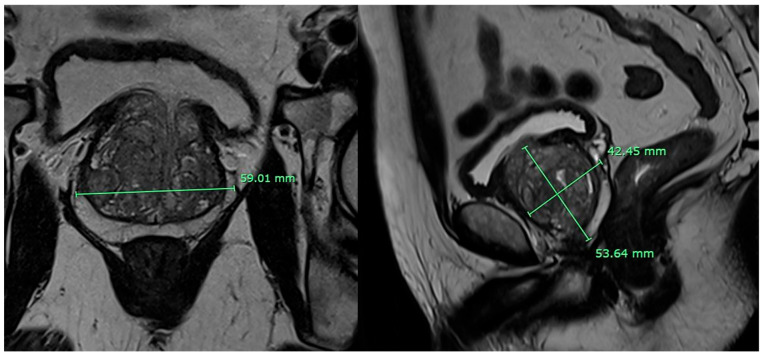
MRI images for prostate volume measurement. Axial T2-weighted image shows the maximum transverse diameter (59.01 mm). Sagittal T2-weighted image demonstrates the superoinferior (53.64 mm) and anteroposterior (42.45 mm) diameters. The calculated prostate volume is approximately 68 mL.

**Multiparametric MRI (mpMRI) protocol:** MRI was acquired on 1.5 T or 3 T systems with a pelvic phased-array coil. The standard PI-RADS v2.1 protocol was followed: high-resolution T2-weighted imaging in axial, sagittal, and coronal planes; diffusion-weighted imaging including high b-values (≥1400 s/mm^2^) with ADC maps; and dynamic contrast-enhanced (DCE) imaging with temporal resolution ≤ 15 s. Lesions were assigned PI-RADS v2.1 scores by subspecialty genitourinary radiologists (≥5 years’ experience) blinded to TRUS results. MRI prostate volume was obtained primarily by contour-based planimetry on axial T2-weighted images (slice-by-slice manual or semi-automated segmentation, defined as software-assisted contouring with automatic boundary detection followed by manual correction by the reader). As a sensitivity analysis, an ellipsoid MRI volume (width × height × length × 0.52) was also recorded when feasible; however, this approach was not applied in cases with irregular gland morphology, poor image quality, or indistinct anatomical boundaries that precluded reliable diameter measurements [30]. Prostate volume was measured on axial and sagittal T2-weighted images using the ellipsoid formula, as illustrated in Figure 3.

The 0.52 ellipsoid constant is widely used across ultrasound and MRI workflows but may introduce bias in irregular gland shapes; several studies suggest ellipsoid underestimates true gland volume compared with reference measures, whereas segmentation can mitigate shape-related error. Accordingly, segmentation was our primary MRI volume method and ellipsoid was a secondary method to evaluate robustness [31,32,33].

PSA density (PSAD) was calculated as total PSA (tPSA, ng/mL) divided by prostate volume (mL) for each modality: TRUS-PSAD and MRI-PSAD. Pre-specified PSAD thresholds used for risk stratification were 0.15 and 0.20 ng/mL/mL, reflecting common cut-points in guidelines and recent literature on MRI-negative men; exploratory analyses also examined 0.30 ng/mL/mL given emerging evidence in selected subgroups, particularly in patients with MRI-negative findings or low-suspicion lesions (e.g., PI-RADS ≤ 3), where higher PSAD thresholds may help reduce unnecessary biopsies [34,35].

When clinically indicated (e.g., PI-RADS ≥ 3, elevated PSAD, or clinician judgment), patients underwent MRI-targeted biopsies (fusion or cognitive) plus systematic 10–12-core sampling (transperineal or transrectal, per local practice) within ≤12 weeks of mpMRI. Biopsy was performed primarily in patients with clinically suspicious findings, including PI-RADS ≥ 3 lesions, elevated PSAD, or clinician judgment. As a result, patients with low-suspicion MRI findings (PI-RADS ≤ 2) were underrepresented in the biopsy cohort. Most biopsies were performed using a combination of MRI-targeted (fusion or cognitive targeting) and systematic 10–12 core sampling, via either transrectal or transperineal approaches according to institutional practice. Prior biopsy status (biopsy-naïve vs. prior negative biopsy) was recorded when available. Histopathology was reported using ISUP Grade Groups. Clinically significant prostate cancer (csPCa) was defined a priori as Grade Group ≥ 2 (Gleason ≥ 3 + 4) (primary definition); sensitivity analyses also considered the PI-RADS v2.1 composite definition (Gleason ≥ 7 and/or tumor volume > 0.5 mL and/or extraprostatic extension) for alignment with imaging literature [29,34,36].

### 2.3. Outcome Measures

Primary outcome: within-person difference in PSAD between modalities (ΔPSAD = MRI-PSAD—TRUS-PSAD), expressed as absolute and relative change, and its clinical impact on biopsy-recommendation reclassification across thresholds (0.15, 0.20; exploratory 0.30). Secondary outcomes: (i) agreement and bias between TRUS and MRI volumes and PSAD; (ii) correlation between volumes/PSAD; (iii) net reclassification improvement (NRI) across thresholds; (iv) diagnostic performance of TRUS-PSAD and MRI-PSAD for csPCa (AUC, sensitivity, specificity); (v) decision curve analysis (DCA) for biopsy strategies using different PSAD thresholds with/without PI-RADS. DCA quantifies net clinical benefit across threshold probabilities and is recommended to complement AUC-based comparisons. For DCA, the combined “MRI-PSAD + PI-RADS ≥ 3” strategy was defined as a conjunctive rule, whereby a patient was considered test-positive only if both MRI-derived PSAD exceeded the predefined threshold and the lesion was classified as PI-RADS ≥ 3.

For reproducibility analysis, a random subset of 50 patients was selected. Two independent radiologists (with ≥5 years of experience in genitourinary imaging) performed MRI-based prostate volume measurements, while two independent sonographers performed TRUS measurements. For inter-observer variability, measurements were performed independently by both readers blinded to each other’s results and to clinical data. For intra-observer variability, one reader from each modality repeated measurements in the same subset after a 4-week interval to minimize recall bias. Inter- and intra-observer reproducibility were assessed using intraclass correlation coefficients (ICC) with 95% confidence intervals, and agreement for PSAD-based categorical thresholds was evaluated using Cohen’s κ statistics. ICC interpretation followed established conventions (poor <0.5; moderate 0.5–0.75; good 0.75–0.9; excellent >0.9). Agreement for dichotomous classification across PSAD thresholds used Cohen’s κ (Landis & Koch benchmarks) [37].

To minimize measurement bias, TRUS operators and MRI readers were blinded to each other’s measurements and to pathology; dimension measurements were standardized (caliper placement landmarks, slice thickness, field-of-view) per PI-RADS v2.1 technical recommendations. Potential spectrum bias (e.g., enrichment for higher PI-RADS categories) was evaluated by reporting the recruitment flow and disease prevalence as recommended by STARD [30].

### 2.4. Statistical Analysis

Statistical analyses were performed using IBM SPSS Statistics v27.0 (IBM Corp., Armonk, NY, USA) and R software (version 4.3.0; R Foundation for Statistical Computing, Vienna, Austria) for advanced analyses. Descriptive statistics were expressed as mean ± standard deviation or median (interquartile range, IQR) for continuous variables, and as frequencies with percentages for categorical variables. Normality was tested using the Shapiro–Wilk test. Paired-sample t-tests or Wilcoxon signed-rank tests were used to compare prostate volumes and PSAD values obtained by TRUS and MRI. Agreement between modalities was assessed by Pearson or Spearman correlation coefficients, as appropriate, and Bland–Altman plots. Reclassification at predefined PSAD thresholds (0.15, 0.20, 0.30 ng/mL/mL) was analyzed using 2 × 2 contingency tables, Cohen’s κ statistics, and Net Reclassification Improvement (NRI). In patients who underwent biopsy, receiver operating characteristic (ROC) curve analysis was performed to assess the diagnostic performance of TRUS-PSAD and MRI-PSAD for csPCa, and the DeLong method was used to compare areas under the curve (AUC). PSAD distributions and csPCa detection rates were reported across PI-RADS categories using descriptive statistics and frequency analysis. Inter- and intra-observer reproducibility for imaging measurements was evaluated using intraclass correlation coefficients (ICC) with 95% confidence intervals, and agreement for dichotomized PSAD thresholds was assessed using Cohen’s κ. Clinical utility of TRUS- and MRI-derived PSAD, alone or combined with PI-RADS, was evaluated using decision curve analysis (DCA) to calculate net clinical benefit across threshold probabilities of 5–30%. A *p*-value < 0.05 was considered statistically significant.

## 3. Results

Baseline characteristics of the study population are shown in Table 1. A total of 202 patients were included in the study, with a mean age of 63.3 ± 7.7 years. The median total PSA level was 8.40 ng/mL (range, 6.59–11.75). The median prostate volume measured by TRUS was 52.5 mL (39.0–72.8), while MRI-derived prostate volume was slightly lower, with a median of 49.0 mL (34.2–70.0). Accordingly, the median TRUS-PSAD was 0.12 (0.09–0.16), compared to 0.17 (0.11–0.25) for MRI-PSAD. The median PI-RADS score was 3 (range, 1–5). Histopathological evaluation revealed benign findings in 138 patients (68.3%), while clinically significant prostate cancer (csPCa; Gleason ≥ 3 + 4) was detected in 64 patients, including 35 (17.3%) with Gleason 3 + 4 and 29 (14.4%) with Gleason > 4 + 3 (Table 1).

Comparison of TRUS and MRI measurements is shown in Table 2. Comparison of prostate volume measurements revealed that MRI-derived volumes were significantly lower than TRUS-derived volumes (median 47.0 mL vs. 52.5 mL, *p* < 0.001). Similarly, PSA density values were higher when calculated using MRI volumes compared with TRUS (median 0.14 vs. 0.12 ng/mL/mL, *p* < 0.001) (Table 2).

**Figure 4:**
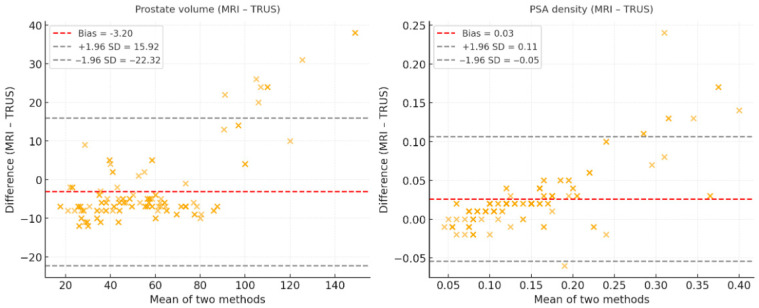
Bland–Altman plots comparing MRI- and TRUS-derived measurements. Prostate volume (MRI–TRUS): The mean difference (bias) and 95% limits of agreement (LoA) are shown. MRI tended to yield slightly smaller volume estimates compared with TRUS. PSA density (MRI–TRUS): MRI-derived PSAD values were slightly higher on average compared with TRUS, with narrow LoA, indicating reasonable agreement.

Bland–Altman analysis demonstrated a negative bias for prostate volume (−3.2 mL) with relatively wide limits of agreement, indicating that MRI tended to underestimate prostate size compared with TRUS. In contrast, MRI-derived PSA density showed a small positive bias (+0.03) with narrower limits of agreement, suggesting better consistency between modalities (Figure 4).

**Figure 5:**
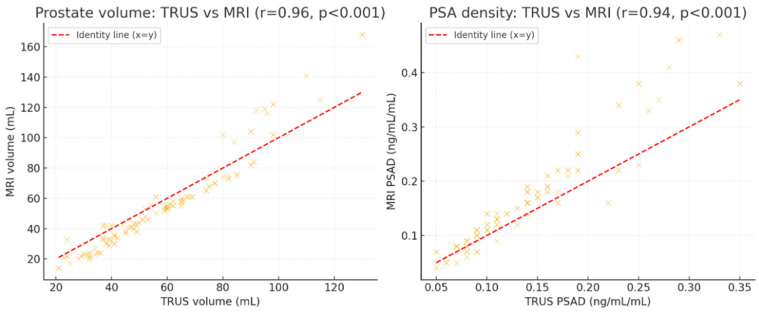
Scatter plots with identity line comparing TRUS- and MRI-derived measurements.

Scatter plot analysis further confirmed strong correlations between TRUS and MRI measurements, with r = 0.96 for prostate volume and r = 0.94 for PSA density (both *p* < 0.001) (Figure 5).

**Figure 6:**
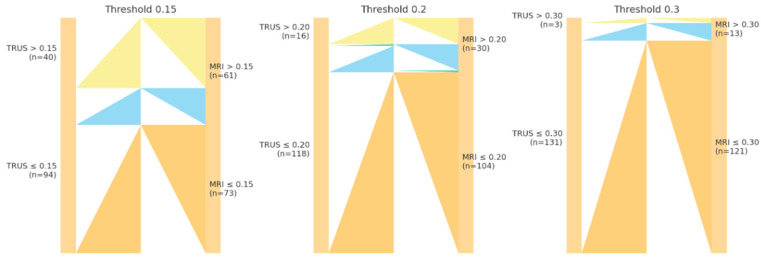
Sankey (alluvial) diagrams illustrating patient reclassification across PSAD thresholds (0.15, 0.20, 0.30 ng/mL/mL) when transitioning from TRUS-derived PSAD to MRI-derived PSAD. Each flow represents the number of patients shifting between negative and positive PSAD categories. Flows from left to right indicate classification based on TRUS-PSAD (left) and MRI-PSAD (right). Upward transitions (negative to positive) reflect risk upgrading with MRI-PSAD, whereas downward transitions (positive to negative) indicate risk downgrading. The diagrams demonstrate that MRI-PSAD more frequently reclassified patients into higher-risk categories, particularly at lower thresholds, contributing to increased sensitivity but reduced specificity.

Reclassification analysis demonstrated notable differences between TRUS- and MRI-derived PSAD values across predefined thresholds. At the 0.15 cutoff, 21 patients classified as negative by TRUS were reclassified as positive by MRI, whereas none shifted from positive to negative; agreement was moderate (κ = 0.675). At the 0.20 cutoff, 15 patients moved from negative (TRUS) to positive (MRI), while 1 patient shifted in the opposite direction (κ = 0.588). At the 0.30 threshold, 10 patients were upgraded by MRI, with no cases downgraded (κ = 0.351), indicating only fair agreement. NRI analyses showed positive reclassification for cancer cases across all thresholds (0.206 at both 0.15 and 0.20; 0.176 at 0.30), though accompanied by negative NRI among non-events, particularly at the 0.15 cutoff (–0.140). Overall, total NRI values were 0.066, 0.136, and 0.136 for thresholds of 0.15, 0.20, and 0.30, respectively. To facilitate interpretation, the Sankey diagrams visualize the direction and magnitude of reclassification, with the majority of flows indicating upward movement from TRUS-negative to MRI-positive categories, particularly at lower PSAD thresholds (Table 3, Figure 6).

**Figure 7:**
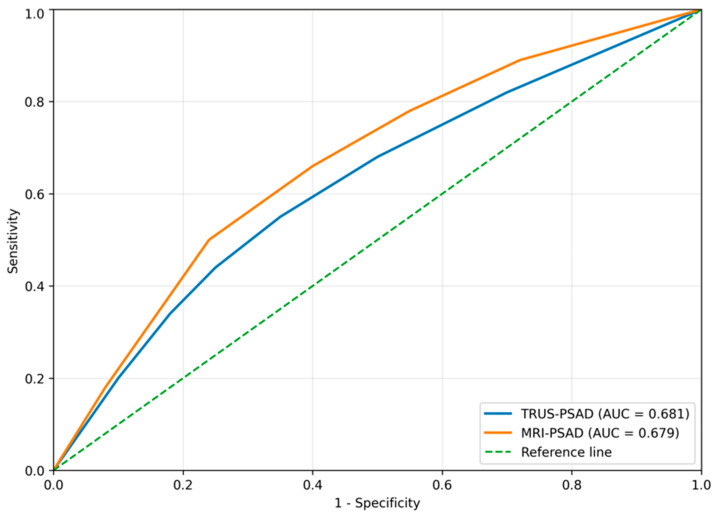
ROC curves comparing TRUS-PSAD and MRI-PSAD for the detection of clinically significant prostate cancer. The curves demonstrate comparable overall discrimination between modalities.

Diagnostic performance of PSA density for clinically significant prostate cancer (csPCA) was shown in Table 4. ROC analysis demonstrated comparable overall diagnostic performance for TRUS-PSAD and MRI-PSAD in detecting clinically significant prostate cancer (csPCa), with similar AUC values (0.681 [95% CI: 0.580–0.770] vs. 0.679 [95% CI: 0.576–0.775]; DeLong *p* = 0.915). At predefined PSAD thresholds, MRI-PSAD consistently showed higher sensitivity but lower specificity compared with TRUS-PSAD. At the 0.15 cutoff, sensitivity increased from 44% (28/64) with TRUS-PSAD to 66% (42/64) with MRI-PSAD, whereas specificity decreased from 75% (104/138) to 60% (83/138). This resulted in a higher negative predictive value for MRI-PSAD (80% vs. 74%) but comparable positive predictive values (43% vs. 45%). At the 0.20 threshold, MRI-PSAD maintained higher sensitivity (45% vs. 25%), with a corresponding reduction in specificity (85% vs. 92%). Positive predictive values were similar between modalities (58% vs. 59%), while MRI-PSAD showed a slightly higher negative predictive value (76% vs. 74%). At the 0.30 cutoff, both modalities demonstrated high specificity, particularly TRUS-PSAD (99% vs. 95%), although MRI-PSAD retained higher sensitivity (22% vs. 6%). Positive predictive values increased at higher thresholds (80% for TRUS-PSAD vs. 67% for MRI-PSAD), while negative predictive values remained comparable (69% vs. 73%) (Table 4, Figure 7).

PSAD distributions and csPCa detection rates varied significantly across PI-RADS categories (Table 5). Patients with PI-RADS 3 lesions (n = 94) had a median TRUS-PSAD of 0.11 (IQR: 0.09–0.16) and MRI-PSAD of 0.14 (IQR: 0.09–0.18), with csPCa confirmed in 9 cases (9.6%). In PI-RADS 4 lesions (n = 36), the median TRUS-PSAD increased to 0.14 (IQR: 0.10–0.18), and MRI-PSAD to 0.16 (IQR: 0.10–0.22), with csPCa detected in 22 patients (61.1%). All PI-RADS 5 cases (n = 3) were diagnosed with csPCa (100%), with higher median PSAD values (TRUS: 0.14, MRI: 0.16). Conversely, the single PI-RADS 2 case showed low PSAD (0.08 by both modalities) and no evidence of csPCa. The distribution of PI-RADS categories reflects a biopsy-selected population, with a predominance of PI-RADS ≥ 3 lesions and minimal representation of low-suspicion (PI-RADS ≤ 2) cases (Table 5).

Inter- and intra-observer reliability for imaging measurements is shown in Table 6. For prostate volume, TRUS demonstrated good agreement with an ICC of 0.86 (95% CI: 0.80–0.91), while MRI achieved excellent reproducibility with an ICC of 0.94 (95% CI: 0.91–0.97). When patients were stratified according to PSAD thresholds, agreement between observers was substantial for TRUS-derived PSAD (κ = 0.71, 95% CI: 0.60–0.82) and almost perfect for MRI-derived PSAD (κ = 0.83, 95% CI: 0.72–0.91) (Table 6).

**Figure 8:**
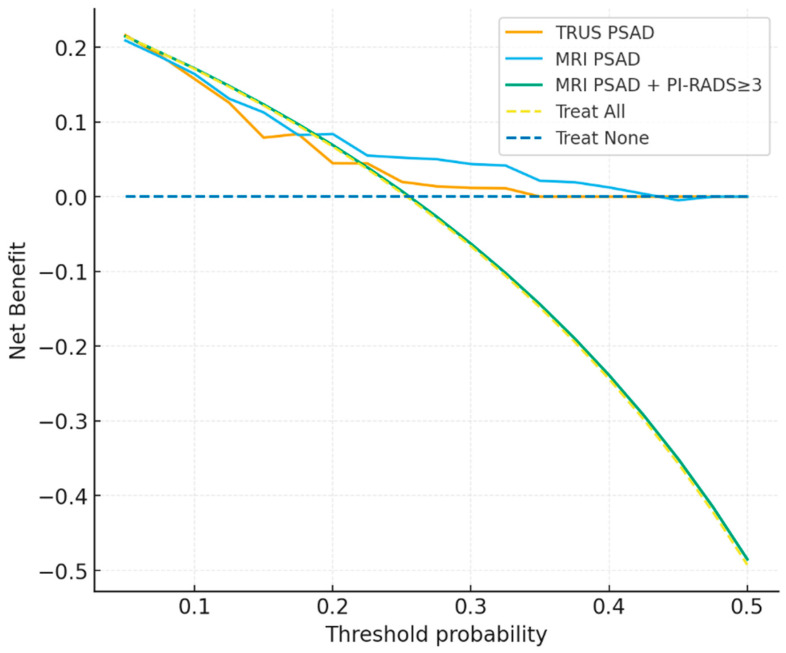
Decision curve analysis (DCA curves: TRUS, MRI, Combination).

The combined strategy required both MRI-PSAD above threshold and PI-RADS ≥ 3 to classify patients as positive. DCA demonstrated that both TRUS- and MRI-derived PSAD provided greater net clinical benefit compared to “treat all” or “treat none” strategies across threshold probabilities of 5–30%. At low to intermediate thresholds (5–15%), MRI-PSAD yielded consistently higher net benefit than TRUS-PSAD (e.g., 0.164 vs. 0.158 at 10%). The combination strategy incorporating MRI-PSAD with PI-RADS ≥ 3 achieved the highest net benefit in this range (0.172 at 10%, 0.123 at 15%). At higher thresholds (20–25%), the difference between TRUS and MRI narrowed, while the added value of the combination strategy decreased (Table 7, Figure 8).

## 4. Discussion

In this study of patients with PSA levels in the diagnostic gray zone, MRI-derived PSA density values were consistently higher than those obtained with TRUS due to systematic differences in prostate volume estimation. Although MRI-PSAD demonstrated higher sensitivity at clinically relevant thresholds, overall diagnostic discrimination between MRI- and TRUS-derived PSAD was comparable, as reflected by nearly identical AUC values. These findings indicate that MRI-based PSAD primarily shifts patients along the same sensitivity–specificity tradeoff curve rather than improving discrimination. Accordingly, the observed differences are better interpreted as threshold-dependent effects rather than true gains in diagnostic performance.

Our Bland–Altman analysis demonstrated that MRI-derived volumes were slightly lower than TRUS measurements (mean bias −3.2 mL), while MRI-PSAD showed a small positive bias (+0.03) with narrower limits of agreement. These results confirm that MRI offers more reliable volume estimation and, consequently, more consistent PSA density values. Mazzone et al. showed that MRI volumetry correlated more closely with prostatectomy specimens and exhibited less inter-observer variability than TRUS [38]. Sonn et al. also highlighted substantial inter-reader variability in MRI interpretation, underscoring the importance of standardized protocols [39]. Collectively, our results support the use of MRI volumetry as a more reproducible and accurate method for PSAD calculation.

Reclassification analyses showed that MRI-PSAD more frequently upgraded patients into higher risk categories, improving sensitivity but reducing specificity. Net Reclassification Improvement confirmed positive reclassification for cancer cases but negative reclassification among non-cancer patients. This trade-off has also been demonstrated by Cobangbang et al., who found that PSAD stratification increased csPCa detection among PI-RADS 4–5 lesions but lowered specificity [40]. Aminsharifi et al. reported that a PSAD cutoff of <0.08 achieved a 96% negative predictive value, reducing unnecessary biopsies at the cost of potentially missing some cancers [41]. Soeterik et al. likewise showed that a cutoff around 0.078 ng/mL/cc maximized sensitivity (~94%) and NPV (~95%), though stricter thresholds risked missed diagnoses [42]. Our findings highlight the importance of selecting clinically appropriate thresholds to balance sensitivity and specificity in gray-zone patients.

Although MRI- and TRUS-derived PSAD yielded nearly identical AUCs, indicating comparable overall diagnostic performance, MRI achieved higher sensitivity at clinically relevant thresholds. MRI-derived PSAD may influence clinical risk categorization by shifting patients across predefined thresholds, primarily due to systematic differences in prostate volume estimation, rather than improving intrinsic diagnostic discrimination. Accordingly, MRI-based PSAD appears to move patients along the existing sensitivity–specificity tradeoff curve without altering overall discrimination. This interpretation is consistent with Luiting et al., who reported that MRI-based PSAD does not substantially improve global discrimination compared with TRUS but may have clinical utility at specific thresholds, particularly in the gray zone [43]. These findings suggest that modality-specific PSAD thresholds may be more appropriate than applying uniform cutoffs across imaging techniques.

Our reproducibility analysis revealed excellent reliability for MRI volumetry (ICC = 0.94) compared with good agreement for TRUS (ICC = 0.86). Agreement for PSAD thresholds was almost perfect for MRI (κ = 0.83) and substantial for TRUS (κ = 0.71). Turkbey et al., who demonstrated that MRI volumetry is less operator-dependent and more reproducible than TRUS, especially when semi-automated segmentation methods are applied [44]. These results strengthen the rationale for MRI as the preferred modality in PSAD-based risk assessment.

Our DCA demonstrated that MRI-PSAD, particularly when combined with PI-RADS ≥ 3, offered the greatest net benefit at lower probability thresholds (5–15%), where the risk of missing csPCa is highest. At higher thresholds (20–25%), the incremental benefit diminished, in line with Schoots et al., who reported that combining PSA density with MRI is most valuable in patients with low-to-moderate suspicion [45]. Rajendran et al. similarly emphasized that integrating MRI-PSAD with PI-RADS improves biopsy decision-making, especially in men with equivocal MRI findings [46]. Together, these findings highlight the clinical role of MRI-PSAD as a key adjunct to PI-RADS for optimizing biopsy strategies and reducing missed diagnoses in gray-zone patients.

Previous studies have extensively explored the role of imaging modality and PSA density in prostate cancer detection, providing important context for our findings. Guo et al. reported that MRI-derived prostate volumes correlated more closely with radical prostatectomy specimen volumes compared with TRUS, confirming the greater accuracy of MRI volumetry [2]. Choe et al. demonstrated in a large cohort that TRUS systematically underestimated prostate volume, while MRI-derived PSAD showed slightly higher odds of detecting csPCa, although AUC differences were modest [24]. Wen et al. showed that in 323 patients with gray-zone PSA, PI-RADS v2.1 alone achieved an AUC of 0.875, PSAD alone was lower (AUC ≈ 0.712), and the combination significantly improved diagnostic performance [47]. Capkan et al. reported that mpMRI-derived parameters, including ADC and PI-RADS scores, were significantly associated with Gleason score in a fusion biopsy-based cohort, supporting the role of MRI in predicting tumor aggressiveness [26]. Aslanoğlu et al. found that PSAD and PI-RADS were complementary; in patients with PI-RADS 4–5 lesions, the cancer detection rate was 54.3%, and combining PSAD ≥ 0.15 with PI-RADS enhanced sensitivity and specificity [1]. Aminsharifi et al. observed that applying higher PSAD thresholds reduced detection of indolent Gleason 6 cancers and MRI use, but at the expense of missing some clinically relevant Gleason ≥ 3 + 4 cases [41]. Aphinives et al. also reported that MRI-PSAD improved both sensitivity and specificity for csPCa detection in men with intermediate PSA levels, supporting our observation that MRI-PSAD more frequently reclassified patients into higher risk categories [25]. In contrast, Kurucz et al. showed that TRUS-based PSAD maintained acceptable accuracy, but tended to overestimate prostate volume and underestimate PSA density relative to MRI [16]. Pantelidou et al. emphasized that while ultrasound-based PSAD remains widely available, MRI-based PSAD offers greater reproducibility and consistency across clinical settings [23]. Collectively, these studies corroborate our results, showing that MRI not only provides more precise volume measurements but also improves risk stratification when integrated into PSA density calculations, particularly in the PSA gray zone.

Our study extends this body of evidence by directly comparing TRUS- and MRI-based PSAD across clinically relevant thresholds and integrating reclassification and decision curve analyses. While most previous studies primarily assessed diagnostic accuracy metrics such as sensitivity and specificity [16,23,24], our results additionally highlight the practical consequences of modality choice on patient classification. MRI-derived PSAD frequently reclassified patients into higher risk categories, leading to improved sensitivity for csPCa detection but at the cost of reduced specificity [25]. Importantly, the use of decision curve analysis provided novel evidence of the net clinical benefit associated with MRI-PSAD, particularly when combined with PI-RADS ≥ 3, emphasizing its potential to reduce missed diagnoses of csPCa in gray-zone populations [28]. By systematically evaluating reclassification, predictive performance, reproducibility, and clinical utility, our study offers a more comprehensive framework for understanding the role of MRI-based PSAD in prostate cancer risk stratification.

From a clinical perspective, our findings highlight the potential of MRI-derived PSAD to improve patient stratification in the gray zone, particularly by reducing missed cases of clinically significant prostate cancer [2,24]. This is consistent with prior fusion biopsy-based studies demonstrating that mpMRI-derived parameters, including ADC values and PI-RADS scores, can support risk stratification and clinical decision-making in prostate cancer [26]. While PI-RADS scoring remains the backbone of MRI-based assessment, adding PSAD may further refine decision-making when imaging results are equivocal [28]. Importantly, clinical application requires careful balancing of sensitivity and specificity to avoid unnecessary biopsies. Future multicenter, prospective studies with larger and more diverse populations are warranted to validate these results and to explore integration of MRI-PSAD with emerging imaging biomarkers, radiomics, or artificial intelligence tools. Such approaches could facilitate personalized diagnostic pathways, reduce overtreatment, and ultimately improve patient outcomes in prostate cancer care.

A major strength of this study is the inclusion of a relatively large cohort of gray-zone patients evaluated with both TRUS and MRI, allowing direct within-patient comparisons. The comprehensive analytical approach—including Bland–Altman agreement analysis, reclassification metrics, ROC analysis, and decision curve evaluation—provides a multidimensional assessment of diagnostic performance and clinical utility. Furthermore, reproducibility was systematically assessed, demonstrating the superior reliability of MRI-derived measurements. Nonetheless, some limitations should be acknowledged. First, this was a single-center retrospective study, which may limit generalizability to broader populations. Second, this study is subject to potential spectrum bias due to the biopsy-selected cohort, in which the majority of patients had PI-RADS ≥ 3 lesions and very few had low-suspicion MRI findings. This limits the generalizability of our results, particularly to MRI-negative or equivocal populations where PSAD may have greater clinical relevance. Therefore, our findings should be interpreted primarily within higher-risk populations undergoing biopsy, and further studies including broader PI-RADS distributions are warranted. Third, inter-observer reproducibility was assessed on a subset of cases rather than the full cohort, and intra-observer variation was not formally analyzed. Finally, while PI-RADS scoring was incorporated into the decision curve analysis, advanced radiomic or machine learning–based risk stratification methods were not evaluated. This cohort composition reflects real-world clinical practice, where biopsy decisions are guided by MRI findings and clinical suspicion.

## 5. Conclusions

In conclusion, in patients with PSA levels in the diagnostic gray zone, MRI-derived PSA density yields systematically higher values compared with TRUS-based measurements due to differences in prostate volume estimation. Although MRI-PSAD demonstrated higher sensitivity at commonly used thresholds, overall diagnostic discrimination between the two modalities was comparable, as reflected by nearly identical AUC values. These findings indicate that differences between MRI- and TRUS-derived PSAD primarily represent threshold-dependent shifts rather than true improvements in diagnostic performance. Therefore, applying modality-specific PSAD cutoffs may be more appropriate than using uniform thresholds across imaging techniques. In clinical practice, MRI-derived PSAD may still be useful when interpreted alongside PI-RADS; however, its added value appears to lie in calibration of decision thresholds rather than enhanced discrimination. Future prospective studies are needed to validate optimal modality-specific cutoffs and their impact on biopsy decision-making.

## Figures and Tables

**Table 1:** Baseline characteristics of the study population (n = 202).

Parameter	Mean ± SD, Median (IQR), n (%)
Age (years)	63.3 ± 7.7
Total PSA (ng/mL)	8.40 (6.59–11.75)
TRUS volume (mL)	52.5 (39.0–72.8)
MRI volume (mL)	49.0 (34.2–70.0)
TRUS PSAD	0.12 (0.09–0.16)
MRI PSAD	0.17 (0.11–0.25)
PI-RADS (median)	3 (1–5)
Histology categories	
Benign	138 (68.3%)
Gleason 3 + 4	35 (17.3%)
Gleason > 4 + 3	29 (14.4%)

**Table 2:** Comparison of TRUS and MRI measurements.

Parameter	TRUS (Median, Min–Max)	MRI (Median, Min–Max)	Wilcoxon Test * (*p* Value)
Prostate volume (mL)	52.5 (21.0–130.0)	47.0 (14.0–168.0)	<0.001
PSA Density (ng/mL/mL)	0.12 (0.05–0.35)	0.14 (0.04–0.47)	<0.001

* Wilcoxon Signed-Rank Test.

**Table 3:** Reclassification between TRUS- and MRI-derived PSAD and net reclassification improvement (NRI).

Threshold	TRUS−/MRI− (n)	TRUS−/MRI+ (n)	TRUS+/MRI− (n)	TRUS+/MRI+ (n)	Cohen’s κ
0.15	73	21	0	40	0.675
0.20	103	15	1	15	0.588
0.30	121	10	0	3	0.351
	**Net Reclassification Improvement (NRI)**				
	**NRI total**	**NRI (events)**	**NRI (non-events)**		
0.15	0.066	0.206	−0.140		
0.20	0.136	0.206	−0.070		
0.30	0.136	0.176	−0.040		

**Table 4:** Diagnostic performance of TRUS-PSAD and MRI-PSAD for clinically significant prostate cancer (csPCa). AUC values are presented with 95% confidence intervals. Sensitivity, specificity, PPV, and NPV are expressed as percentages and were calculated based on biopsy-confirmed csPCa.

Threshold/Metric	TRUS PSAD		MRI PSAD	
AUC (95% CI)	0.681 (0.580–0.770)		0.679 (0.576–0.775)	
	**Sensitivity**	**Specificity**	**Sensitivity**	**Specificity**
0.15	44% (28/64)	75% (104/138)	66% (42/64)	60% (83/138)
0.20	25% (16/64)	92% (127/138)	45% (29/64)	85% (117/138)
0.30	6% (4/64)	99% (137/138)	22% (14/64)	95% (131/138)
	**PPV**	**NPV**	**PPV**	**NPV**
0.15	45%	74%	43%	80%
0.20	59%	74%	58%	76%
0.30	80%	69%	67%	73%
**DeLong test**	*p* = 0.915			

**Table 5:** PSA density and clinically significant prostate cancer (csPCa) rates by PI-RADS category (v2.1 protocol).

PI-RADS	n	TRUS PSAD	MRI PSAD	csPCa n	csPCa %
		(Median, IQR)			
2	1	0.08 (0.08–0.08)	0.08 (0.08–0.08)	0	0.0%
3	94	0.11 (0.09–0.16)	0.14 (0.09–0.18)	9	9.6%
4	36	0.14 (0.10–0.18)	0.16 (0.10–0.22)	22	61.1%
5	3	0.14 (0.14–0.24)	0.16 (0.16–0.32)	3	100.0%

**Table 6:** Inter- and intra-observer reliability for imaging measurements.

Measurement Type	Modality	Analysis Type	ICC (95% CI)	κ (95% CI)
Prostate volume	TRUS	Inter-observer	0.86 (0.80–0.91)	–
Prostate volume	MRI	Inter-observer	0.94 (0.91–0.97)	–
Prostate volume	TRUS	Intra-observer	0.89 (0.83–0.93)	–
Prostate volume	MRI	Intra-observer	0.96 (0.93–0.98)	–
PSAD threshold	TRUS	Inter-observer	–	0.71 (0.60–0.82)
PSAD threshold	MRI	Inter-observer	–	0.83 (0.72–0.91)
PSAD threshold	TRUS	Intra-observer	–	0.76 (0.65–0.86)
PSAD threshold	MRI	Intra-observer	–	0.88 (0.78–0.94)

ICC: Intraclass correlation coefficient; κ: Cohen’s kappa. Inter-observer agreement was assessed between two independent readers. Intra-observer agreement was evaluated by repeated measurements performed by the same reader after a 4-week interval.

**Table 7:** Decision curve analysis (DCA) (TRUS vs MRI vs Combination).

Threshold Probability	Net Benefit TRUS PSAD	Net Benefit MRI PSAD	Net Benefit MRI PSAD + PI-RADS ≥ 3
5%	0.216	0.209	0.214
10%	0.158	0.164	0.172
15%	0.079	0.113	0.123
20%	0.045	0.084	0.069
25%	0.020	0.052	0.007

## Data Availability

The datasets used and/or analyzed during the current study are available from the corresponding author upon reasonable request.

## Abbreviations

PSA	Prostate-Specific Antigen
PSAD	Prostate-Specific Antigen Density
TRUS	Transrectal Ultrasound
MRI	Magnetic Resonance Imaging
mpMRI	Multiparametric Magnetic Resonance Imaging
PI-RADS	Prostate Imaging-Reporting and Data System
csPCa	Clinically Significant Prostate Cancer
NRI	Net Reclassification Improvement
ROC	Receiver Operating Characteristic
AUC	Area Under the Curve
ICC	Intraclass Correlation Coefficient
LoA	Limits of Agreement
NPV	Negative Predictive Value
PPV	Positive Predictive Value
DCA	Decision Curve Analysis
IQR	Interquartile Range
ISUP	International Society of Urological Pathology
